# Differential spatio-temporal regulation of MMPs in the 5xFAD mouse model of Alzheimer’s disease: evidence for a pro-amyloidogenic role of MT1-MMP

**DOI:** 10.3389/fnagi.2014.00247

**Published:** 2014-09-18

**Authors:** Nathalie A. Py, Amandine E. Bonnet, Anne Bernard, Yannick Marchalant, Eliane Charrat, Frédéric Checler, Michel Khrestchatisky, Kévin Baranger, Santiago Rivera

**Affiliations:** ^1^Faculty of Medicine, Aix-Marseille Université, CNRS, NICN, UMR7259Marseille, France; ^2^IPMC UMR 7275 CNRS-UNS, Labex DistAlzValbonne, France; ^3^Department of Neurology and Neuropsychology, APHM, CHU La TimoneMarseille, France

**Keywords:** metalloproteinases, amyloid, MMP-2, MMP-9, ADAM, TIMP, neuroinflammation, cytokines

## Abstract

Matrix metalloproteinases (MMPs) are pleiotropic endopeptidases involved in a variety of neurodegenerative/neuroinflammatory processes through their interactions with a large number of substrates. Among those, the amyloid precursor protein (APP) and the beta amyloid peptide (Aβ) are largely associated with the development of Alzheimer’s disease (AD). However, the regulation and potential contribution of MMPs to AD remains unclear. In this study, we investigated the evolution of the expression of MMP-2, MMP-9, and membrane-type 1-MMP (MT1-MMP) in the hippocampus at different stages of the pathology (asymptomatic, prodromal-like and symptomatic) in the 5xFAD transgenic mouse AD model. In parallel we also followed the expression of functionally associated factors. Overall, the expression of MMP-2, MMP-9, and MT1-MMP was upregulated concomitantly with the tissue inhibitor of MMPs-1 (TIMP-1) and several markers of inflammatory/glial response. The three MMPs exhibited age- and cell-dependent upregulation of their expression, with MMP-2 and MMP-9 being primarily located to astrocytes, and MT1-MMP to neurons. MMP-9 and MT1-MMP were also prominently present in amyloid plaques. The levels of active MT1-MMP were highly upregulated in membrane-enriched fractions of hippocampus at 6 months of age (symptomatic phase), when the levels of APP, its metabolites APP C-terminal fragments (CTFs), and Aβ trimers were the highest. Overexpression of MT1-MMP in HEK cells carrying the human APP Swedish mutation (HEKswe) strongly increased β-secretase derived C-terminal APP fragment (C99) and Aβ levels, whereas MMP-2 overexpression nearly abolished Aβ production without affecting C99. Our data consolidate the emerging idea of a regulatory interplay between MMPs and the APP/Aβ system, and demonstrate for the first time the pro-amyloidogenic features of MT1-MMP. Further investigation will be justified to evaluate this MMP as a novel potential therapeutic target in AD.

## Introduction

Alzheimer’s disease (AD) is the most common and devastating neurodegenerative disorder. Histopathologically, AD is characterized by the accumulation of intracellular and extracellular deposits of amyloid beta peptide (Aβ), which according to the amyloid cascade hypothesis is considered as a crucial process in AD pathogenesis (Hardy and Higgins, 1992; Karran et al., 2011). Amyloid beta peptide results from the sequential cleavage of the amyloid precursor protein (APP) by β- and γ-secretases. Its clearance is in part mediated by Aβ-degrading metalloproteinases, including neprilysin (NEP), insulin-degrading enzyme (IDE), angiotensin-converting enzyme (ACE), endothelin-converting enzyme (ECE) and matrix metalloproteinases (MMPs; De Strooper, 2010; Rivera et al., 2010; Chami and Checler, 2012). Matrix metalloproteinases constitute a family of ~25 zinc-dependent endopeptidases produced by all neural cell types. They interact with a wide range of substrates (e.g., extracellular matrix proteins, cytokines, growth factors, cell adhesion molecules, receptors), which confer to MMPs broad functional diversity (Rivera et al., 2010). They are thus involved in numerous pathological and physiological processes: neural cell motility and outgrowth (Ogier et al., 2006; Gonthier et al., 2007; Ould-yahoui et al., 2009), neuronal death (Jourquin et al., 2003), synaptic plasticity and cognition (Jourquin et al., 2005; Chaillan et al., 2006; Meighan et al., 2006; Nagy et al., 2006; Kaliszewska et al., 2012), neuroinflammation (Ogier et al., 2005; Hu et al., 2007; Candelario-Jalil et al., 2009) and blood brain barrier (BBB) breakdown (Yang et al., 2007).

Several MMPs, including MMP-2, MMP-9 and membrane-type 1-MMP (MT1-MMP), a principal activator of MMP-2, may degrade Aβ (Roher et al., 1994; Backstrom et al., 1996; Yin et al., 2006; Liao and Van Nostrand, 2010). In return, Aβ upregulates the expression of MMP-2 and/or MMP-9 in cultured neurons, (Mizoguchi et al., 2009), astrocytes (Deb et al., 2003), and neuroblastoma cells (Talamagas et al., 2007). MT1-MMP expression is upregulated in cultured human cerebrovascular smooth muscle cells upon prolonged treatment with pathogenic concentrations of Aβ (Jung et al., 2003). Moreover, MMP-2 and MT1-MMP have been suggested to cleave APP *in cellulo* and generate soluble truncated APP forms with yet unknown functions (LePage et al., 1995; Higashi and Miyazaki, 2003; Ahmad et al., 2006). *In vivo*, MMP-2 and MMP-9 expression is upregulated in reactive astrocytes of the Tg2576 transgenic mouse model of AD at advanced stages of the pathology concurrent with massive amyloid deposition (Yin et al., 2006). However, the evolution of MMPs expression from asymptomatic to symptomatic phases is unknown, and yet it may provide valuable insights on the role of these proteinases in AD. From previous work (Oakley et al., 2006; Jawhar et al., 2012; Giannoni et al., 2013; Girard et al., 2013), the 5xFAD mouse model of AD has been shown to recapitulate within the first 6 months of life asymptomatic, prodromal-like and symptomatic phases reminiscent of AD pathology. Accordingly, we have investigated the brain distribution of MMP-2, MMP-9 and MT1-MMP at 2, 4 and 6 months, each time point corresponding to these three phases, respectively. Our data clearly indicate specific patterns of spatio-temporal distribution for these MMPs along with changes in inflammatory factors and Aβ-degrading enzymes. Our study also reveals that the level of MT1-MMP increases with age in 5xFAD hippocampus, in pace with the increase of APP and its C-terminal fragments (CTFs), and Aβ trimers. We found *in cellulo* that MT1-MMP overexpression enhanced the β-secretase-derived CTF (C99) and Aβ production, and thus conclude that MT1-MMP could be a new pro-amyloidogenic proteinase and a novel target in AD pathogenesis.

## Methods

### Animals

Experiments were performed in transgenic hemizygous 5xFAD male mice and their wild-type (WT) littermates (Jackson Laboratories, Bar Harbor, ME, USA) on a C57BL6/SJL background. 5xFAD mice harbor three familial Alzheimer’s disease (FAD) mutations in the human *APP*695 (Swedish K670N, M671L; Florida I716V and London V717I) and two mutations in the human presenilin-1 (*PSEN-1*) (M146L, L286V) under the transcriptional control of the neuron-specific mouse Thy-1 promoter. These mice show over a 6- to 9-month period intracellular and extracellular accumulation of Aβ42, gliosis, synaptic demise, cognitive impairment and neuronal loss (Oakley et al., 2006; Ohno et al., 2007; Girard et al., 2013). Behavioral dysfunctions have been primarily described in 5xFAD females (Jawhar et al., 2012), which present a more prominent amyloid pathology than males. However, recent behavioral data obtained using olfactory-based tests, have uncovered subtle deficits of cortical- and hippocampal-dependent cognition in males as early as 4 months that are absent at 2 months and are more severe at 6 months (Girard et al., 2013, 2014). Another study has also highlighted hippocampal-dependent spatial memory deficits in 5-month-old 5xFAD males in the Morris water maze (Hongpaisan et al., 2011). Taken together, these data indicate an age-dependent progression of functional deficits in 5xFAD mice that affect the hippocampus and roughly correspond to the asymptomatic, prodromal-like and symptomatic phases of the pathology at 2, 4 and 6 months of age, respectively. Accordingly, these time points were chosen to investigate the evolution of MMPs and other molecular systems in 5xFAD males. Procedures were conducted in accordance with National and European regulations (EU directive N° 2010/63) and in agreement with the authorization for animal experimentation attributed to the laboratory by the Prefecture des Bouches du Rhône (permit number: D 13 055 08). All efforts were made to minimize animal suffering and to reduce the number of mice used.

### Genotyping

Newborn pups were genotyped by polymerase chain reaction (PCR). Genomic DNA was isolated from tail biopsies using 60 μL of lysis buffer (25 mM NaOH, 0.2 mM EDTA) at 100°C for 30 min. Samples were then cooled to −20°C for 5 min and 60 μL of 40 mM Tris-HCl buffer was added to neutralize the lysis reaction. Five μL of DNA were amplified with *PSEN-1* primers by a classic PCR reaction: denaturation for 5 min at 95°C, followed by 40 cycles each consisting of 94°C for 3 min, 55°C for 45 s and 72°C for 1 min (Jackson Laboratories). PCR products were separated by electrophoresis in a 1.5% agarose gel containing ethidium bromide and visualized under UV light lamp.

### Plasmid constructions

MT1-MMP and MMP-2 cDNAs were amplified by PCR from P15 C57Bl6 mice cerebellum mRNA, and cloned and expressed as previously reported for other MMP cDNAs (Sbai et al., 2008, 2010; Ould-yahoui et al., 2009). The following forward (For) and reverse (Rev) primers were used: *MT1For* AAT TAT GGA TCC CGG ACC TTG TCC AGC AGC GAA C, *MT1Rev* TAT ATA CTC GAG AGG AGA GCA GAG AGG GCT TC, *MMP-2For* ATA TAT GAA TTC GCC AGA GAC CTC AGG GTG ACA CGC and *MMP-2Rev* ATA TAT GTC GAC AGG CAG CCC AGC CAG TCT GAT TTG AT. All constructs were cloned into pEGFP-N1 (Clontech, Saint-Germain-en-Laye, France). Plasmids coding for GFP-, MT1-MMP, and MMP-2 were amplified in *E. coli* DH5α (Life Technologies, Saint Aubin, France) and purified using the NucleoBond Xtra Midi Plus EF (Macherey-Nagel, Hoerd, France) according to manufacturer’s recommendations.

### HEKswe cell culture and Aβ production

HEK cells stably transfected with pcDNA3 vector overexpressing human APP harboring the double Swedish mutation (HEKswe) were used (Chevallier et al., 1997). Cells were plated to 1 × 10^6^ cells/mL for 24 h in 6-well plates in DMEM Glutamax, FBS 10%, penicillin/streptomycin 1% (Life Technologies). The cells were transfected with 1 μg of plasmids coding for MT1-MMP/GFP or MMP-2/GFP fusion proteins using the Jet Pei transfection reagent (Ozyme, Saint-Quentin en Yvelines, France), as previously described (Sbai et al., 2010). Twelve hours after transfection, the medium was replaced with OptiMEM containing 1% ITS (Life Technologies) and cells were allowed to secrete for 48 h. Human Aβ40 peptide levels were measured in the culture supernatants using an ELISA assay (Life Technologies).

### Reverse transcription quantitative polymerase chain reaction (RT-QPCR)

Total RNA was extracted from mice hippocampi using the Nucleospin RNA II kit (Macherey-Nagel) according to the manufacturer’s instructions. cDNA was synthesized from 500 ng of RNA using random primers (Life Technologies) and Moloney Murine Leukemia Virus Reverse Transcriptase (M-MLV RT, Life Technologies) in a total volume of 50 μL. Twenty-five ng of cDNA were submitted to qPCR reaction. All the probes used in this study are represented in Table 1. *Gapdh* gene expression was used as an endogenous control for evaluation of all mRNA levels. qPCR was performed using the Fast Real-Time PCR System according to the manufacturer’s recommendations (Applied Biosystems, Life Technologies). For each experiment, four different cDNA samples were analyzed in duplicate. Relative gene expression was obtained using the comparative 2^−(ΔΔCt)^ method after normalization to the *Gapdh* housekeeping gene.

**Table 1:** **Summary of TaqMan mouse probes used for quantitative PCR**.

Gene name	Gene description	Probes ID
*Mmp-2*	Matrix metalloproteinase 2	Mm 00439498-m1
*Mmp-9*	Matrix metalloproteinase 9	Mm 00442991-m1
*Mt1-mmp*	Membrane-type 1 matrix metalloproteinase	Mm 00485054-m1
*Timp-1*	Tissue inhibitor of matrix metalloproteinase 1	Mm 00441818-m1
*Timp-2*	Tissue inhibitor of matrix metalloproteinase 2	Mm 00441825-m1
*Timp-3*	Tissue inhibitor of matrix metalloproteinase 3	Mm 00441826-m1
*Timp-4*	Tissue inhibitor of matrix metalloproteinase 4	Mm 00446568-m1
*Adam-10*	A disintegrin and metalloproteinase 10	Mm 00545742-m1
*Adam-17*	A disintegrin and metalloproteinase 17	Mm 00456428-m1
*Nep*	Neprilysin	Mm 00485028-m1
*Ace*	Angiotensin-converting enzyme	Mm 00802048-m1
*Ece*	Endothelin-converting enzyme	Mm 01187091-m1
*Ide*	Insulin-degrading enzyme	Mm 00473077-m1
*Il1b*	Interleukin-1 beta	Mm 01336189-m1
*Ccl2*	Chemokine ligand 2	Mm 00441242-m1
*Gfap*	Glial fibrillary acidic protein	Mm 01253033-m1
*Emr1*	F4/80, marker for mouse macrophages	Mm 00802529-m1
*Gapdh*	Glyceraldehyde-3-phosphate dehydrogenase	Mm 99999915-g1

*Probes are provided by Applied Biosystems*.

### Immunohistochemistry

Mice were anesthetized with pentobarbital (0.36 g/kg) and transcardially perfused with 50 mL of NaCl 0.9% followed by 50 mL of 4% paraformaldehyde (PFA). Mice brains were then removed and post-fixed for 24 h in PFA at 4°C. Coronal sections (30 μm thick) were obtained using a vibratome (Microm HM 650 V, Thermo scientific, MA, USA), and stored at −20°C in cryoprotectant containing 30% glycerol, 30% ethylene glycol and 40% phosphate buffer 0.5 M pH 7.4. Free-floating sections were first permeabilized and binding blocked for 1 h at room temperature using a solution of PBS 1X, 0.1% Triton X-100 and 3% Bovine Serum Albumin (BSA, Sigma-Aldrich, Saint Quentin-Fallavier, France). Sections were then incubated overnight at 4°C with primary antibodies (see Table 2), followed by Alexa 488-goat anti-rabbit (1/500) or Alexa 594-goat anti-mouse (1/500) (both from Life Technologies) for 1 h at room temperature. Nuclei were stained with Hoechst (0.5 μg/mL, Life Technologies). Omission of the primary antibody was used as control and no immunostaining was observed. Sections were mounted using Prolong Gold Antifading reagent on Superfrost glass slides (Life Technologies). Images were taken and processed using a confocal microscope (LSM 700) and Zen software (Zeiss, Jena, Germany).

**Table 2:** **Detail of primary and secondary antibodies used for western blot and immunohistochemistry in this study**.

		Dilution				Dilution	
Antigens	Species	WB	IHC	Company	2nd Antibody	WB	IHC
MMP-2	Rabbit	1/250	1/200	Millipore	Goat anti Rabbit	1/1000	1/500
MMP-9	Rabbit	1/500	1/200	Millipore	Goat anti Rabbit	1/1000	1/500
MT1-MMP	Rabbit	1/500	1/100	Abcam	Goat anti Rabbit	1/500	1/500
TIMP-1	Goat	1/300	x	R8D systems	Donkey anti Goat	1/500	x
TIMP-2	Mouse	1/1000	x	Abcam	Goat anti Mouse	1/2000	x
APP 22C11	Mouse	1/500	x	Millipore	Goat anti Mouse	1/2000	x
Aβ 6E10	Mouse	1/300	1/300	Covance	Goat anti Mouse	1/500	x
APP-CTF	Rabbit	1/2000	x	Sigma	Goat anti Rabbit	1/4000	x
GFP	Mouse	1/1000	x	Roche	Goat anti Mouse	1/3000	x
GFAP	Mouse	x	1/200	Millipore	Goat anti Mouse	x	1/500
MAP-2	Chicken	x	1/200	Abcam	Donkey anti Chicken	x	1/500
GAPDH	Mouse	1/2000	x	Millipore	Goat anti Mouse	1/8000	x
Actin	Mouse	1/5000	x	Sigma	Goat anti Mouse	1/10000	x

*Horse radish peroxydase (HRP) coupled secondary antibodies (Jackson Immunoresearch) were used for western blot (WB). Alexa-coupled secondary antibodies (Invitrogen) were used for immunohistochemistry (IHC)*.

### *In situ* zymography

*In situ* zymography was performed as previously described, with slight modifications (Jourquin et al., 2005). After anesthesia and NaCl perfusion (see above), the brain was removed from the skull and snap frozen in cold isopentane. Coronal brain sections (30 μm thick) were obtained with a cryostat (CM 3050 LEICA, Germany), collected on Superfrost Plus® slides and stored at −80°C. Brain sections were incubated with 20 μg/ml FITC-gelatin (Life Technologies) for 1 h at 37°C in a dark humidified chamber, rinsed in PBS, fixed with PFA for 30 min, and immunostained with 6E10 specific anti-Aβ antibody (see Table 2). Images were analyzed as described above.

### Western blotting

After NaCl transcardial perfusion, the hippocampi were microdissected and snap-frozen for biochemical assays. For western blots (WB), each sample was homogenized in 25% w/v of 50 mM Tris-HCl pH 7.5 buffer containing 150 mM NaCl, 2 mM EDTA, 1% Triton X-100, 0.05% SDS and proteinase inhibitor cocktail (Millipore, Molsheim, France) and centrifuged at 10,000× *g* for 20 min to obtain the “soluble fraction”. Cell pellets were resuspended in 25% w/v of 50 mM Tris-HCl pH 7.5 buffer containing 2% SDS, then sonicated and centrifuged at 10,000× *g* for 20 min to obtain the insoluble “membrane-enriched fraction”. Protein concentrations were determined using a Bio-Rad *DC^TM^* protein assay kit (Bio-Rad, Marnes-La-Coquette, France), and 50 μg of protein were run on 10% to 15% SDS-PAGE gels and transferred to nitrocellulose membranes (Amersham Bioscience, Velizy-Villacoublay, France). After blocking, membranes were probed with primary antibodies, as indicated in Table 2 and then incubated with the corresponding horseradish peroxidase-conjugated secondary IgG antibodies (Jackson Immunoresearch, Suffolk, UK). Immunodetection was performed using the ECL kit according to the manufacturer’s instructions (GE Healthcare, Dutscher, Brumath, France), and optical density measured using the imageJ software (NIH). All optical density plots represent normalized values to actin or GAPDH as indicated in the figure legends.

### Statistics

All values represent the means ± SEM of the number of animals or independent cultures indicated in the figure legends. Student-*t* test was used to compare two groups. ANOVA analysis followed by a Tukey’s *post hoc* test was used to compare more than two groups. Statistical significance was set up to *p* < 0.05. Analyses were performed with the Kaleidagraph software (Synergy Software, Reading PA, USA).

## Results

### Expression of metalloproteinases, TIMPs and inflammatory markers in the hippocampus of 5xFAD mice across age

**Figure 1:**
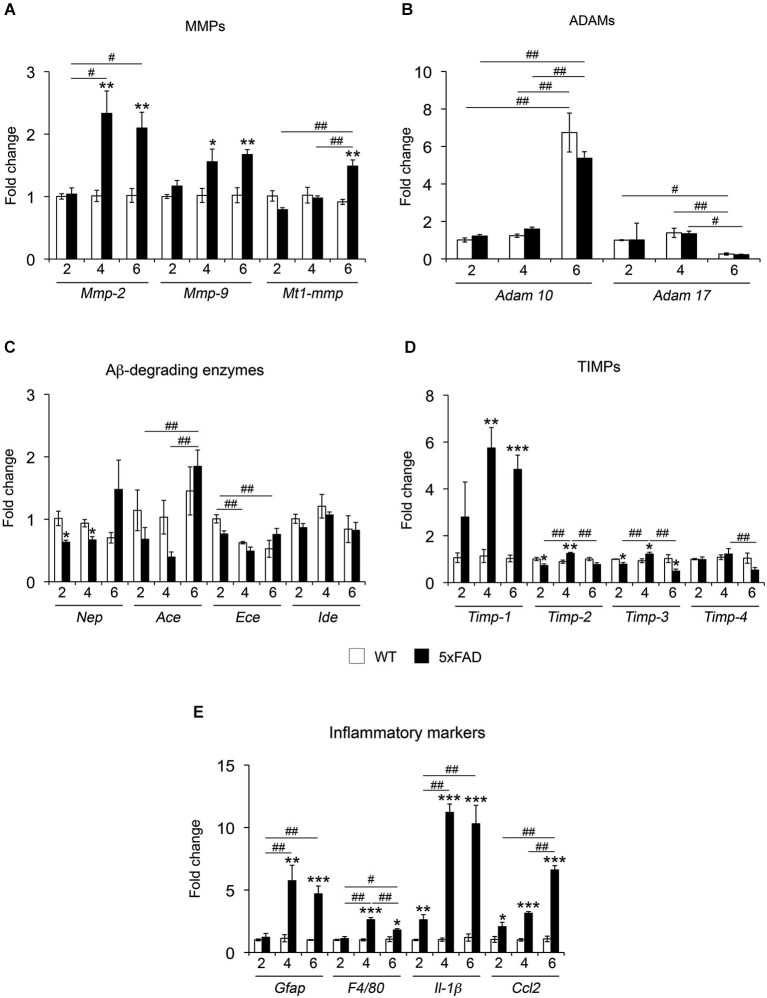
**mRNA expression of different proteolytic systems and inflammatory markers across age in 5xFAD mice hippocampi**. mRNA expression for MMPs **(A)** ADAMs **(B)** Aβ-degrading enzymes **(C)**, TIMPs **(D)** and inflammatory markers **(E)** was analyzed in the hippocampus of WT and 5xFAD mice at 2, 4 and 6 months. Values are the mean ± SEM of four mice per group representing fold change differences between genotypes. *p < 0.05, **p < 0.01, ***p < 0.001 when comparing 5xFAD *vs*. WT of the same age; ^#^*p* < 0.05, ^##^*p* < 0.01, ^###^*p* < 0.001 when comparing different ages of the same genotype; ANOVA followed by Tukey’s test.

To obtain a broad picture of the regulation of metalloproteinases potentially involved in Aβ/APP metabolism across different phases of the amyloid pathology, we assessed the mRNA levels of MMPs, adamalysins 10 and 17 (ADAM-10 and -17) and classical Aβ-degrading enzymes (NEP, IDE, ACE, ECE). In addition, we also measured the mRNA levels of tissue inhibitor of MMPs (TIMPs), the natural inhibitors of MMPs and ADAMs, and a number of prototypic inflammatory markers (Figures 1A–E). Among MMPs, the expression of *Mmp-2* and *Mmp-9* remained unaffected in 2-month-old mice and was significantly increased 50% and 130% in 5xFAD mice compared to WT at 4 and 6 months, respectively (Figure 1A). *Mt1-mmp* showed a significant increase of 50% only at 6 months. In contrast, the levels of *Adam-10* and *Adam-17* remained stable between genotypes, but increased and decreased respectively at 6 months as compared to previous time points for the same genotype (Figure 1B). The expression of major Aβ-degrading enzymes did not differ significantly between 5xFAD and WT mice, with the exception of *Nep*, which showed significant decreases (30%) at 2 and 4 months (Figure 1C). Among the four TIMPs, *Timp-1* exhibited strong 4 and 5-fold increases at 4 and 6 months, respectively. The expression of *Timp-2* significantly decreased at 2 months and increased at 4 months. The level of *Timp-3* mRNA decreased at 2 and 6 months while it increased at 4 months. No differences were observed between WT and 5xFAD for *Timp-4* (Figure 1D). Specific markers of astrocyte (*Gfap)* and microglia (*F4/80)* reactivity remained stable at 2 months and significantly increased at 4 and 6 months. Furthermore, the levels of the pro-inflammatory mediators interleukin-1beta (*Il-1β)* and chemokine ligand 2 (*Ccl2*) (also known as monocyte chemoattractant protein-1 (*Mcp-1*), were already upregulated 2.6-fold in 5xFAD brains as early as 2 months of age, illustrating an early inflammatory response in these mice before the appearance of the first cognitive deficits. By 4 and 6 months, *Il-1β* levels reached a 12-fold increase compared to WT. *Ccl2* reached a peak of expression by 6 months (7-fold) (Figure 1E). Overall, the MMP/TIMP system seems to be more affected (mostly upregulated) than the ADAMs or the Aβ-degrading enzyme subfamilies. The upregulation of MMP and TIMP levels occur at 4 months of age, coincident with the upregulation of markers of glial reactivity and in the context of an ongoing upregulation of inflammatory markers that precedes changes in all other genes.

### MMPs and TIMPs content in the hippocampus of 5xFAD and WT mice across age

**Figure 2:**
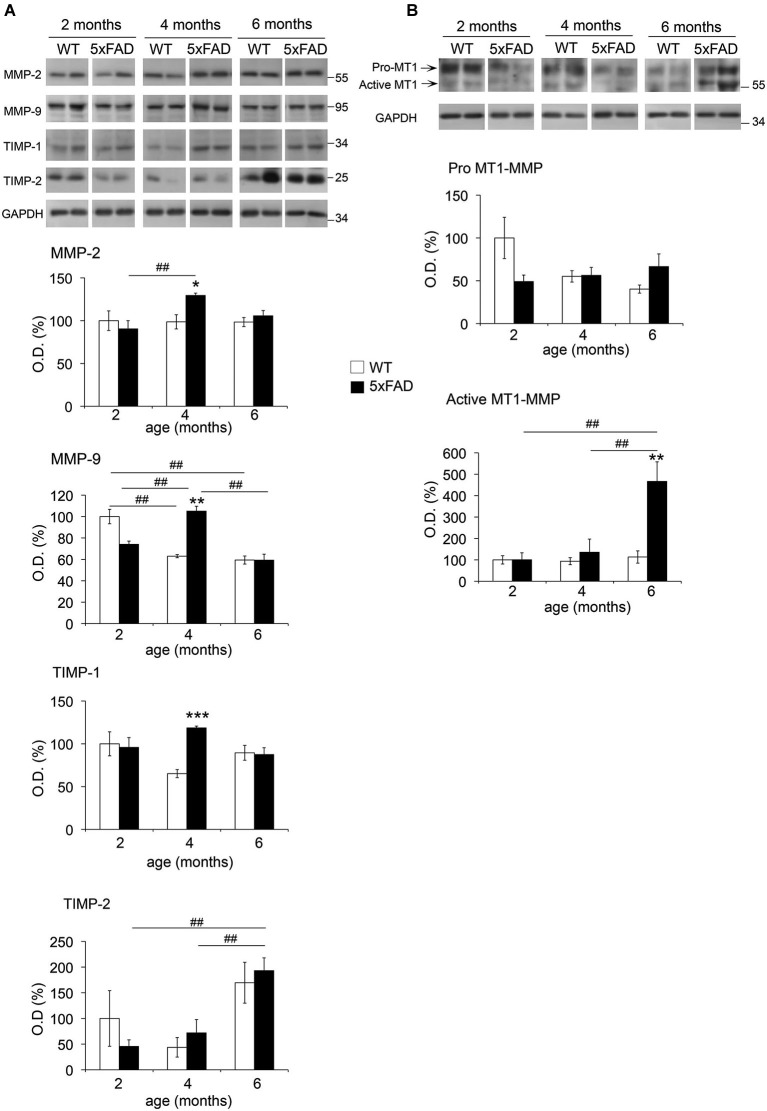
**Age-dependent changes in MMPs and TIMPs across age in 5xFAD mice hippocampi**. Western blots and the corresponding quantifications of MMP-2, MMP-9, TIMP-1 and TIMP-2 in the soluble fraction **(A)**, and of MT1-MMP pro- and active forms in the membrane-enriched fraction **(B)** of WT and 5xFAD mice hippocampi at 2, 4 and 6 months of age. GAPDH normalized values are the mean ± SEM of four mice per group representing the percentage of variation in optical density (O.D.). *p < 0.05, **p < 0.01, ***p < 0.001 when comparing 5xFAD *vs*. WT of the same age; ^#^*p* < 0.05, ^##^*p* < 0.01, ^###^*p* < 0.001 when comparing the same genotype across age; ANOVA followed by Tukey’s test.

MMP levels from hippocampal soluble and membrane-enriched fractions were analyzed by WB. In the soluble fraction, MMP-2 levels did not change with age in WT mice, whereas MMP-9 exhibited a significant decrease (35%) between 2 and 4–6 months (Figure 2A). In 5xFAD mice, the levels of both MMPs increased significantly at 4 months of age compared to 2 months. For MMP-9, this increase was also significant when compared to 6 months. Moreover, we found that MMP-2 and MMP-9 levels were increased in 5xFAD compared to WT only at 4 months by a 30% and 67%, respectively. We observed no changes in MMP-2 and MMP-9 levels between genotypes at 2 or 6 months. An additional faint band (~90 kDa) compatible with the expected size of the active form of MMP-9 was also detected (Figure 2A), but no significant differences were found across genotypes (data not shown).

Two immunoreactive bands for anti-MT1-MMP were observed at ~65 kDa and ~55 kDa in the membrane-enriched fraction (Figure 2B), coincident with the expected size of pro-MT1-MMP and active MT1-MMP, respectively. No significant changes were observed for pro-MT1-MMP across age or between genotypes. In contrast, active MT1-MMP levels in 5xFAD mice increased 4.5-fold at 6 months compared to 2 and 4 months, and also to WT at 6 months. In the soluble fraction, where MT1-MMP was barely detectable, we did not evidence changes in its expression across age or between genotypes (data not shown).

TIMP-1, a major endogenous inhibitor of secreted MMPs and ADAMs (e.g., ADAM-10), presented 83% higher levels at 4 months in 5xFAD compared to WT hippocampi. Neither WT nor 5xFAD mice showed significant differences in TIMP-1 content across age. No differences between genotypes were detected in the levels of TIMP-2, which inhibits MT-MMPs and also secreted MMPs. However, TIMP-2 exhibited a remarkable 3-fold increase in 5xFAD hippocampi at 6 months compared to 2 and 4 months. It is noteworthy that TIMPs were not detected in the insoluble fraction (data not shown).

### Comparison of MMP-2 expression in the hippocampus of WT and 5xFAD mice

**Figure 3:**
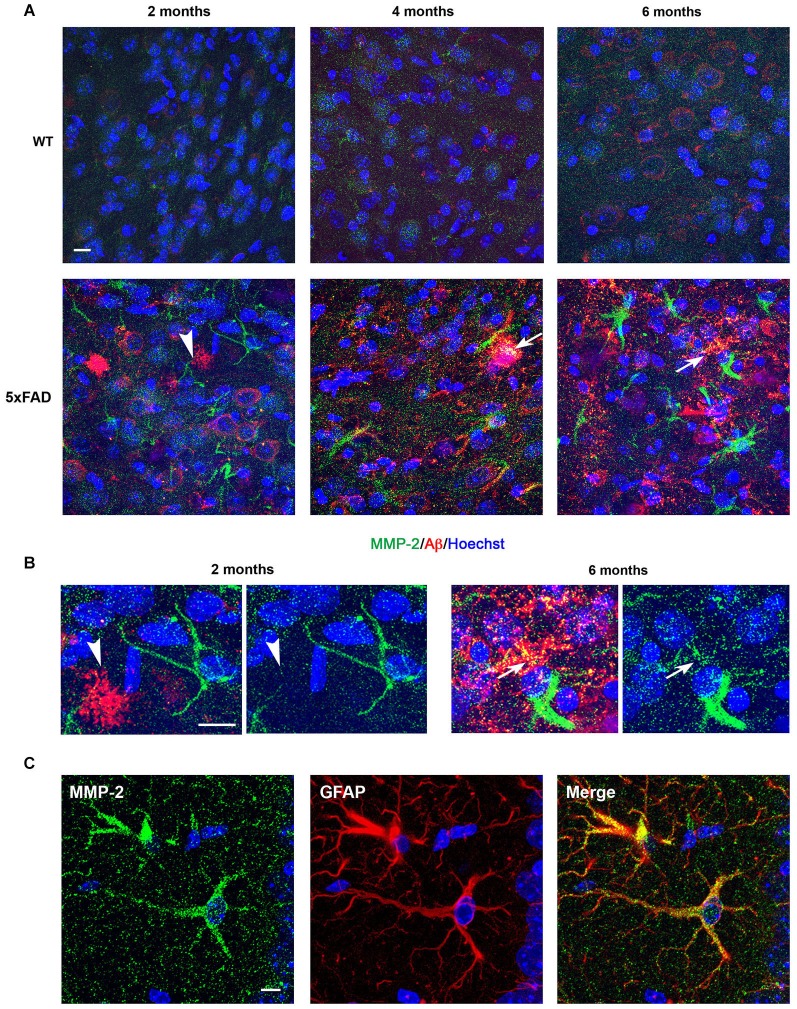
**Age-dependent upregulation of MMP-2 expression in astrocytes and amyloid plaques in 5xFAD mice hippocampi**. **(A)** Confocal microphotographs representative of four animals per group showing combined immunohistochemistry with the Aβ specific 6E10 antibody (red) and a MMP-2 specific antibody (green) in the subiculum of WT and 5xFAD mice at 2, 4 and 6 months of age. Note that MMP-2 is upregulated in neural cells with astrocyte morphology in the vicinity of amyloid plaques (arrowhead) as early as 2 months. MMP-2 immunostaining increases with age in 5xFAD mice, and at 4 and 6 months there is partial colocalization with amyloid plaques, as revealed by yellow color (arrows). **(B)** High power magnifications of 5xFAD *subiculum* at 2 and 6 months showing non-association and association of MMP-2 with amyloid deposits, respectively. Hoechst (blue) labels cell nuclei. **(C)** Confocal microphotographs showing localization of MMP-2 (green) to GFAP+ astrocytes (red) of 5xFAD mice. Scale bars: 10 μm.

To further assess the cellular distribution of MMP-2, MMP-9 and MT1-MMP, coronal brain sections from WT and 5xFAD mice were immunolabeled using specific MMP antibodies. As illustrated in the *subiculum*, weak MMP-2 immunoreactivity was diffusely distributed in WT hippocampi at 2 months of age (Figure 3A, upper left). In contrast, MMP-2 staining in 5xFAD brains was present in glial cells with astrocyte morphology in the vicinity of incipient amyloid deposits (Figure 3A, lower left). MMP-2 immunostaining steadily increased at 4 and 6 months in glial cells associated with larger and more numerous amyloid plaques, especially in the hippocampal *subiculum*, where amyloid plaques were mainly concentrated (Figure 3A, lower panels). No MMP-2 immunoreactivity was observed in amyloid plaques at 2 months. At 4 and 6 months, most amyloid plaques exhibited moderate immunoreactivity for MMP-2 (Figure 3B). Double immunostaining using anti-MMP-2 and anti-GFAP antibodies confirmed the astrocytic expression of MMP-2 (Figure 3C).

### Comparison of MMP-9 expression in the hippocampus of WT and 5xFAD mice

**Figure 4:**
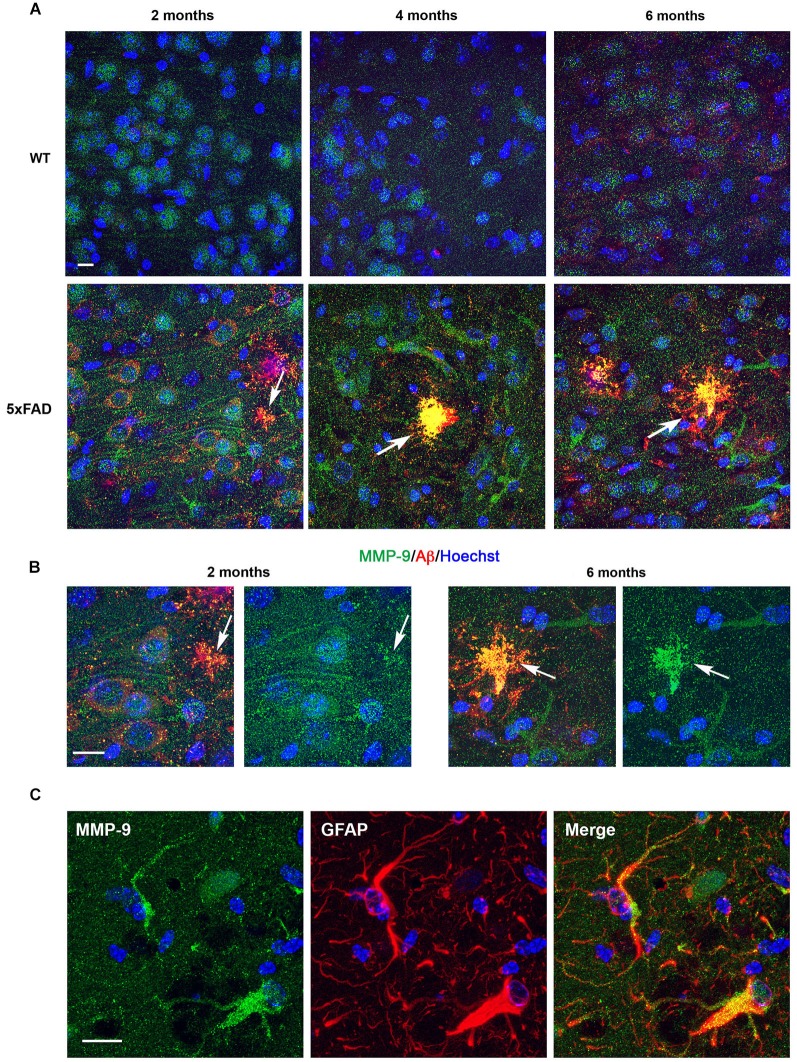
**Age-dependent upregulation of MMP-9 expression in astrocytes and amyloid plaques in 5xFAD mice hippocampi**. **(A)** Confocal microphotographs representative of four animals per group showing combined immunohistochemistry with the Aβ specific 6E10 antibody (red) and a MMP-9 specific antibody (green) in the *subiculum* of WT and 5xFAD mice at 2, 4 and 6 months of age. Note that at 2 months MMP-9 presents a somato-dendritic distribution in 5xFAD neurons, in contrast with WT where it is essentially somatic. Also, at 2 months MMP-9 shows incipient localization to amyloid plaques, as revealed by yellow color (arrows). MMP-9 immunostaining increases with age in astrocytes of 5xFAD mice, and at 4 and 6 months colocalization with amyloid plaques is noticeable. Hoechst (blue) labels cell nuclei. **(B)** High power magnifications of 5xFAD *subiculum* at 2 and 6 months showing age-dependent increased association of MMP-9 with amyloid deposits. **(C)** Confocal microphotographs showing localization of MMP-9 (green) to GFAP+ astrocytes (red) of 5xFAD mice. Scale bars: 10 μm.

In the *subiculum* of 2-month-old WT mice, MMP-9 immunostaining was confined to neuronal cell bodies (Figure 4A, upper left). In age-matched 5xFAD mice, MMP-9 immunoreactivity was detected in both soma and dendrites (Figure 4A, lower left). By 4 and 6 months, MMP-9 immunostaining in 5xFAD mice was mainly present in reactive astrocytes, whereas we could no longer detect dendrite-associated immunoreactivity (Figure 4A, lower middle and right panels). Unlike MMP-2, MMP-9 colocalized with 6E10 immunostaining in amyloid plaques as early as 2 months, and immunostaining was very prominent in these amyloid structures at 4 and 6 months (Figure 4B). Double immunostaining using anti-MMP-9 and anti-GFAP antibodies confirmed the astrocytic expression of MMP-9 in particular at 4 and 6 months of age (Figure 4C).

### Comparison of MT1-MMP expression in the hippocampus of WT and 5xFAD mice

**Figure 5:**
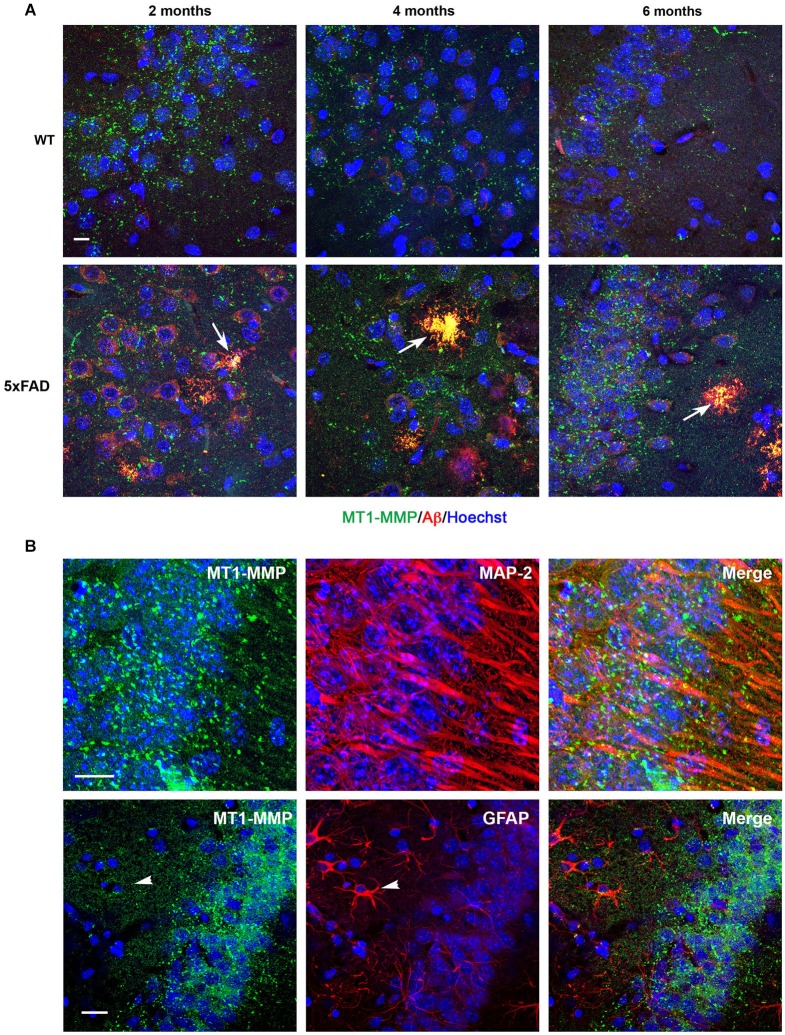
**Age-dependent upregulation of MT1-MMP expression in neurons and amyloid plaques in 5xFAD mice hippocampi**. **(A)** Confocal microphotographs representative of four animals per group showing combined immunohistochemistry with the Aβ specific 6E10 antibody (red) and a MT1-MMP specific antibody (green) in the *subiculum* of WT and 5xFAD mice at 2, 4 and 6 months of age. MT1-MMP presents a punctate distribution in the neuronal cell layer. MT1-MMP immunostaining increases at 6 months in 5xFAD hippocampi and colocalizes with amyloid plaques at all ages studied, as revealed by yellow color (arrows). Hoechst (blue) labels cell nuclei. **(B)** High power magnifications of 5xFAD *subiculum* showing localization of MT1-MMP (green) to MAP-2+ neuronal cell bodies (red, upper panels), and no colocalization with GFAP+ astrocytes (red, lower panels). Scale bars: 10 μm.

In WT hippocampi, MT1-MMP immunostaining presented a punctate distribution that localized to neuronal cell layers at all ages studied (Figure 5A). In the 5xFAD mice, the intensity of labeling correlated with the density of amyloid load. Thus, subicular neurons exhibited more prominent MT1-MMP signal than other areas in the hippocampus (data not shown). Like, MMP-9, MT1-MMP immunostaining was already present in amyloid plaques at 2 months, and this colocalization persisted across time (Figure 5A, lower panels). Double immunostaining with anti-MAP-2 specific neuronal marker confirmed that MT1-MMP immunolabeling localized preferentially to neuronal soma (Figure 5B, upper panels). Double immunostaining with anti-GFAP antibodies confirmed that MT1-MMP was not expressed by astrocytes (Figure 5B, lower panels).

### MMP-dependent gelatinolytic activity is present in amyloid plaques

**Figure 6:**
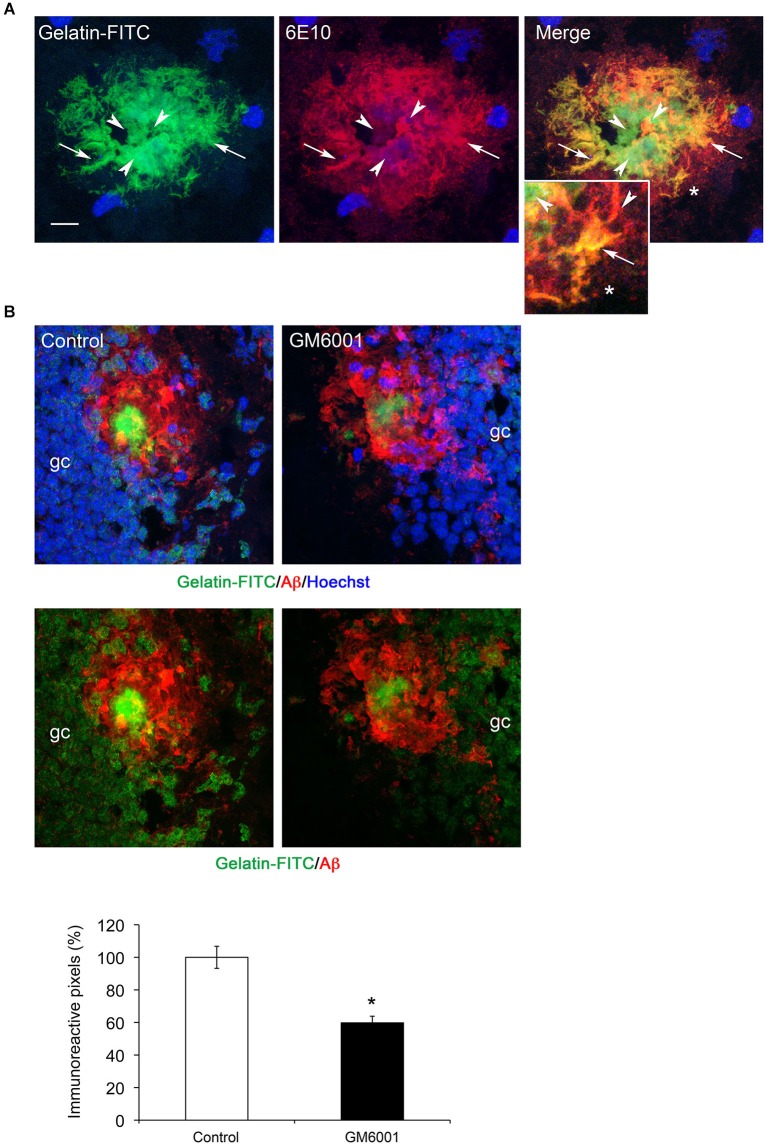
**MMP gelatinolytic activity is present in amyloid plaques. (A)** Confocal microphotographs representative of four animals per group showing combined immunohistochemistry using the Aβ specific 6E10 antibody (red) and gelatin-FITC *in situ* zymography (green) in the hippocampus of 6-month-old 5xFAD mouse. Note that zones of perfect colocalization (arrows, yellow color) coexist with zones of poor or non-colocalization (arrowheads), often associated with dense core amyloid. **(B)** Confocal microphotographs showing reduction of gelatinolytic activity (green) in plaques (6E10, red) and granule cells (gc) in the dentate gyrus of 5xFAD mice at 6 months. Lower panels without Hoechst staining show that gelatinolysis is reduced in the amyloid plaque and in granule cells. The graph shows a ~50% reduction in gelatin-FITC signal after GM6001 incubation. Values are the mean ± SEM of four mice per group. *p < 0.05; Student *t*-test.

A common feature to MMP-2, MMP-9 and MT1-MMP in the present study is their presence in extracellular amyloid deposits. The three MMPs have the ability to degrade gelatin (Imai et al., 1996; Rivera et al., 2002), with MMP-2 and MMP-9 being more efficient than MT1-MMP. Thus, we used gelatin-based *in situ* zymography to determine the distribution of gelatinolytic activity with respect to Aβ deposits. We found strong gelatinolytic activity in the hippocampus preferentially associated with amyloid plaques. Gelatin-FITC signal was generally more intense in plaque areas with diffuse amyloid than in those with a dense core amyloid (Figure 6A, see arrows). Incubation of fresh brain slices with GM6001, a broad spectrum MMP inhibitor, inhibited fluorescence signal about 50% (Figure 6B), indicating an important contribution of MMPs to proteolytic activity in amyloid plaques.

### Age-dependent increase in the levels of APP, APP-CTFs and Aβ trimers in 5xFAD mice

**Figure 7:**
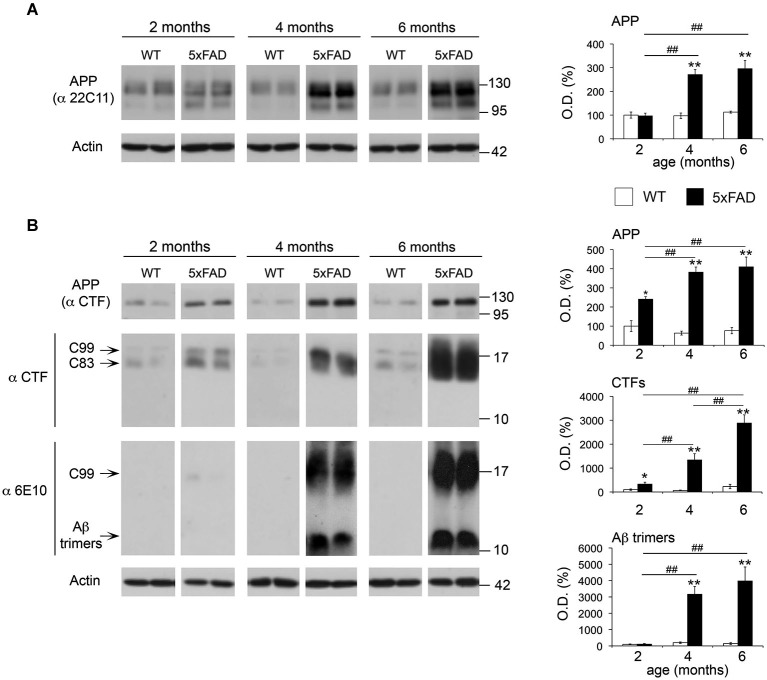
**Age-dependent upregulation of APP, APP CTFs and Aβ trimers in 5xFAD hippocampi**. **(A)** Western blot showing full-length APP levels and the corresponding quantification, using the 22C11 antibody on the soluble fraction of WT and 5xFAD hippocampi at 2, 4 and 6 months. **(B)** Western blot and the corresponding quantifications of full-length APP, APP CTFs (C99 and C83) and Aβ trimers using the CTF and 6E10 antibodies on membrane-enriched fractions of the same animals. Actin normalized values are the mean ± SEM of four mice per group representing the percentage of variation in optical density (O.D.). *p < 0.05, **p < 0.01, when comparing 5xFAD *vs*. WT of the same age; ^#^*p* < 0.05, ^##^*p* < 0.01, when comparing the same genotype across age; ANOVA followed by Tukey’s test.

A progressive increase of soluble and fibrillar Aβ across time has been previously described in 5xFAD brains (Oakley et al., 2006). However, we have limited information on the age-dependent changes in APP and its metabolites APP-CTFs, and Aβ oligomers. Gaining insight into such evolution may be important to support a pathophysiological role of APP metabolites and to decipher possible functional links with MMPs depending on their spatio-temporal distribution. In this context, the β-secretase-derived CTF of APP, C99 (βCTF) and small Aβ oligomers (dimers and trimers) have been shown to be particularly neurotoxic (Walsh et al., 2002; Cleary et al., 2005; Lesné et al., 2006; Townsend et al., 2006; Shankar et al., 2008; Lauritzen et al., 2012). As illustrated in Figure 7, APP levels increased by 2.6-fold in the soluble fraction of 5xFAD hippocampi at 4 and 6 months of age compared to WT, as revealed by the 22C11 antibody directed against the N-terminal domain of APP (Figure 7A). In the membrane-enriched fraction, the APP C-terminal specific CTF antibody revealed a significant 3.8-fold increase of full length APP, as early as 2 months of age, and 5.4-fold increase by 4 and 6 months (Figure 7B). Concomitantly, we found a dramatic upregulation of β- and α-CTFs (C99 and C83, respectively) compared to WT at 2 (3.3-fold), 4 (20-fold) and 6 (13-fold) months. In the same membrane-enriched fraction, the human Aβ specific 6E10 antibody demonstrated between 15- and 30-fold increase in the levels of putative Aβ trimers of ~12 kDa in 5xFAD mice at 4 and 6 months, compared to background levels in the WT. It is noteworthy that the ~12 kDa band was not detected in the membrane enriched fractions at 2 months (Figure 7B) nor in the soluble fractions, regardless of age (data not shown). The absence of the ~12 kDa band in the CTF immunoblot, together with its detection by the 6E10 antibody, reinforced the idea that the latter indeed detected Aβ trimers and not a CTF of APP containing the 6E10 epitope located between the β- and α-cleavage sites. These data show a clear correspondence between the upregulation of APP, CTFs and Aβ trimers in the same membrane-enriched fractions where the levels of active MT1-MMP were also increased.

### MT1-MMP overexpression increases C99 and Aβ levels in HEKswe cells

**Figure 8:**
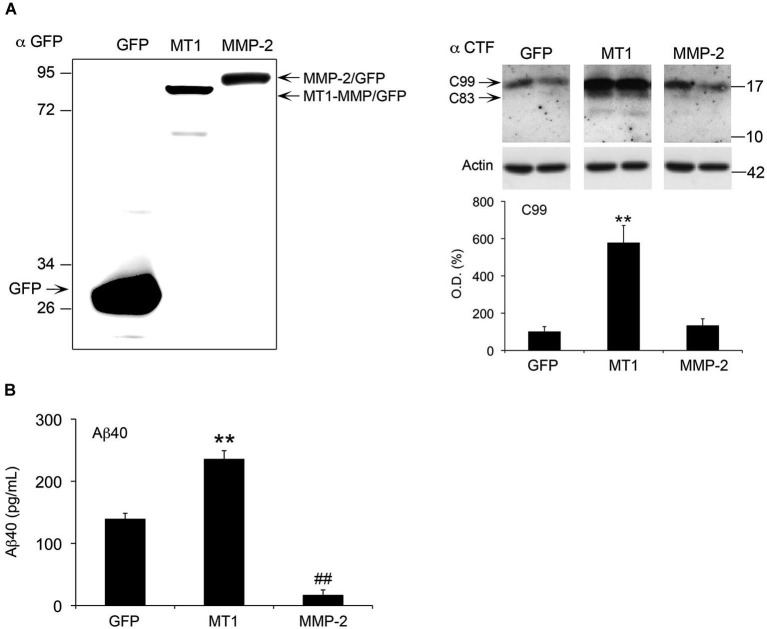
**MT1-MMP and MMP-2 have opposite roles in the regulation of C99 and Aβ in HEKswe cells**. **(A)** Left panel, western blot using a GFP specific antibody illustrates a representative experiment showing equivalent levels of overexpression for MT1-MMP/GFP (MT1) and MMP-2/GFP fusion proteins 48 h post-transfection. Right panel, western blot with the corresponding quantification of APP C99 levels, using the CTF antibody on whole cell lysates of HEKswe cells 48 h after transfection of plasmids coding for GPF, MT1-MMP/GFP and MMP-2/GFP fusion proteins. Actin normalized values are the mean ± SEM of four independent cultures representing the percentage of variation in optical density (O.D.). **p < 0.01, MT1 *vs*. GFP and MMP-2; ANOVA followed by Tukey’s test. **(B)** ELISA assay showing opposite effects on Aβ40 levels in the supernatant of HEKswe cells 48 h post-transfection. MT1-MMP significantly induced Aβ production, whereas MMP-2 nearly abolished it. Values are the mean ± SEM of four independent cultures. **p < 0.01, MT1 *vs*. GFP and MMP-2; ^##^*p* < 0.01, MMP-2 *vs*. GFP. ANOVA followed by Tukey’s test.

The coincidence between increased levels of active MT1-MMP, and increased APP metabolism and CTFs accumulation prompted us to investigate if MT1-MMP could contribute to generate these APP CTFs. To address this point, we transiently expressed a MT1-MMP/GFP fusion protein in HEKswe cells stably expressing APP with the Swedish mutation that is also present in 5xFAD mice. In parallel, we also expressed a MMP-2/GFP fusion protein in these cells. We found that MT1-MMP/GFP induced a 6-fold increase in the levels of a 17 kDa band detected by the CTF antibody compatible with the size of C99 (Figure 8A). A faint band immediately below, likely representing C83, was also induced by MT1-MMP/GFP. In contrast with the marked increase in C99 levels induced by MT1-MMP, the overexpression of MMP-2/GFP did not affect CTF levels, which remained close to GFP control values. Since C99 is the substrate of γ-secretase to produce Aβ, we next investigated whether increased C99 levels would impact Aβ formation after MT1-MMP overexpression in HEKswe cells. The latter overproduce Aβ40 with respect to Aβ42 and only the former is easily detected (Buggia-Prevot et al., 2008). Moreover, it is known that Aβ42 forms oligomers much faster than does Aβ40 (Soreghan et al., 1994). Together, a low Aβ42/Aβ40 ratio, combined with a relatively short post-transfection period may compromise the detection of Aβ oligomers in these cells. Accordingly, we used a sensitive quantitative ELISA assay to assess global soluble levels of Aβ40 as a suitable readout of amyloidogenesis. We found that MT1-MMP-mediated increase of C99 was concomitant with a 70% increase of Aβ40 levels in the supernatants of HEKswe cells (Figure 8B). On the opposite, MMP-2/GFP overexpression nearly abolished Aβ40 production, which was decreased 88% with respect to GFP control. Together, these data clearly underline the amyloidogenic properties of MT1-MMP and the anti-amyloidogenic activity of MMP-2 (Figure 8B).

## Discussion

The present study describes specific patterns of expression for several MMPs at different stages of Alzheimer’s pathology in the 5xFAD mouse model. We extend previously reported data on advanced stages of the pathology in other mouse models to show that expression of MMP-2 and MMP-9 is mainly upregulated in reactive astrocytes and amyloid plaques, as early as 2 months of age and further confirmed at later stages (4 and 6 months), suggesting their importance in the evolution of the amyloidogenic process. Most interestingly, our data uncover the upregulation of the active form of MT1-MMP in the 5xFAD hippocampus at 6 months, along with strong accumulation of APP C-terminal fragment C99 and Aβ trimers. This starts at 4 months of age, which seems to signal a stage of the pathology where APP metabolism is suddenly accelerated in 5xFAD hippocampi. We finally demonstrated in HEKswe cells that MT1-MMP overexpression increased the production of both Aβ and its precursor C99, suggesting a possible link between increased MT1-MMP activity *in vivo* and amyloidosis. In summary, we provide evidence that three MMPs potentially involved in APP/Aβ metabolism are differentially upregulated across age in a transgenic mouse model of AD, and among them, MT1-MMP may be a new pro-amyloidogenic proteinase.

### The mRNA expression of proteolytic systems and inflammatory markers is differentially regulated across the different phases of the pathology in 5xFAD mice.

The three stages studied underline correlations between the progression of Aβ load and pahtology. Our qPCR study reveals that pro-inflammatory markers previously associated with AD (e.g., *Il-1β*, and *Ccl2*) are the only genes significantly upregulated at 2 months, possibly signifying the early response of the system to incipient Aβ production. The age-dependent increases in mRNA expression for *Il-1β* and *Ccl2*, associated with the increase of markers of astrocyte (*Gfap*) and microglial (*F4/80*) reactivity further comforts the widely assumed idea of a regulatory link between Aβ production and neuroinflammation. In this context, the proteolytic systems studied displayed rather differential profiles. While mRNA levels of major α-secretases and Aβ-degrading enzymes remained basically unaffected, MMPs were overall upregulated in 5xFAD mice compared to WT. Such selective modulation of MMP gene expression may result from the prominent ability of pro-inflammatory cytokines to stimulate MMPs (Fanjul-Fernández et al., 2010; Rivera et al., 2010). Seemingly, TIMPs are also modulated by cytokines, in particular TIMP-1, whose expression is significantly upregulated by Il-1β (Dafnis et al., 2012). Accordingly, TIMP-1 expression increased in 5xFAD hippocampi at 4 months and thereafter, in pace with the upregulated expression of its main targets MMP-9 and MMP-2. While some members of the MMP/TIMP system clearly showed upregulation of mRNA expression, the expected translation into increased protein levels was not explicitly observed. This is particularly evident for TIMP-1, whose mRNA levels were dramatically increased at 4 and 6 months in 5xFAD mice, while protein levels were only significantly upregulated at 4 months. This apparent inconsistency at 6 months could reflect adaptive mechanisms (e.g., increased turnover of TIMP-1) set in motion to keep the levels of TIMP-1 within a physiological range, and therefore prevent for instance excessive inhibition of metalloproteinases presumably involved in AD homeostatic responses; α-secretase ADAM-10 and Aβ-degrading enzymes MMP-2 and MMP-9 are paradigmatic examples of TIMP-1 targets (Rivera et al., 2010). The implementation of feedback mechanism of control between protein and mRNA may be feasible in chronic situations such as AD, clearly in contrast with acute brain insult (i.e., excitotoxic seizures), where rapid and transient upregulation of TIMP-1 mRNA is followed by the subsequent increase of TIMP-1 protein levels (Rivera et al., 1997).

### Differential cell-dependent upregulation of MMPs across age

Our study confirms in 5xFAD mice previous work reporting the increase of MMP-2 and MMP-9 immunostaining in reactive astrocytes around plaques in the APP/PS1 transgenic mouse model of AD (Yin et al., 2006). The same study suggested that the release of these MMPs by astrocytes contribute to Aβ degradation. Interestingly, despite the well-reported activation of microglial cells in 5xFAD mice (Oakley et al., 2006; Girard et al., 2013), neither MMP-2 nor MMP-9 seemed to be associated with these cells. In the case of MMP-9, its microglial upregulation has been previously associated with neurotoxic effects on hippocampal neurons (Rivera et al., 2002; Jourquin et al., 2003), indirectly suggesting that activated microglia in the 2- to 6-month old 5xFAD mice are involved in beneficial rather than detrimental processes. By combining *in situ* zymography and MMP inhibition with GM6001, we further confirmed MMP-dependent gelatinolytic activity especially in amyloid plaques. Since MMP-9 and MT1-MMP immunostaining were particularly prominent in amyloid plaques, it is possible that they contribute to gelatinolysis in a significant manner, which would be consistent with the singular ability of both MMPs to degrade fibrillar Aβ (Yan et al., 2006; Liao and Van Nostrand, 2010). The pathophysiological consequences of amyloid plaque degradation by these MMPs have not been investigated yet, and will likely depend on the cytotoxicity of the potentially released breakdown products (e.g., oligomers). In our study, we found upregulation of MMP-2 and MMP-9 levels (mRNA and protein) at 4 months, while no differences were observed between WT and 5xFAD in 2- and 6-month-old mice. This is in contrast with the maintaining of important MMP levels in reactive astrocytes and amyloid plaques, and may signify a global overall increase around the prodromal phase of the pathology, coincident with an important activation of APP metabolism. Indeed, at 4 months of age we observe a dramatic increase of CTFs, soluble Aβ and Aβ oligomers. The transient increase of MMPs with Aβ-degrading activity may reflect the homeostatic response of the organism to the increase of Aβ load. In the long term, a sustained increase of these MMPs may not be compatible with the preservation of neural function. In this context, MMP-9, in particular, has been shown to be neurotoxic for hippocampal neurons (Jourquin et al., 2003) and both MMP-2 and MMP-9 may disturb BBB function and foster the inflammatory response (Yang et al., 2007).

Besides amyloid plaques, MT1-MMP was primarily associated with neurons. Previous studies reported astrocytic MT1-MMP expression in the thalamus of 24-month-old Tg-SW DI mice, a model where the localization of fibrillar Aβ deposits is restricted to cerebral microvessels (Liao and Van Nostrand, 2010). Another study suggested the expression of MT1-MMP in reactive microglial cells around amyloid plaques in 5xFAD mice slightly older than those used in the present work and in postmortem AD brains (Langenfurth et al., 2013). Our double immunostaining experiments failed to establish any colocalization of MT1-MMP with cells other than neurons in the hippocampus over the time period studied. Differences in the time points, the immunostaining protocols or the stage of the pathology studied in these different works might account for such cell-type differences. The temporal MT1-MMP profile of expression differed with respect to MMP-2 and MMP-9. Indeed, MT1-MMP upregulation occurred in the membrane-enriched fraction during the symptomatic phase of the pathology at 6 months, as demonstrated by WB. Increases were observed in the active form of MT1-MMP without significant changes in the content of its precursor pro-MT1-MMP. We conclude from these data that the production of pro-MT1-MMP likely increases at 6 months, but this is efficiently cleaved by the intracellular proprotein convertase furin to generate the active form (Mazzone et al., 2004). Although the mechanism that triggers MT1-MMP upregulation at 6 months remains to be elucidated, one possibility is that the progressive age-dependent accumulation of Aβ and/or APP stimulates the production of MT1-MMP. This appears to be the case when human cerebrovascular smooth muscle cells are treated with Aβ (Jung et al., 2003) or after overexpression of APP in HEK cells (Ahmad et al., 2006).

### APP and its toxic metabolites increase with age in 5xFAD mice

Not surprisingly, the expression of the human transgene upregulated the levels of APP in 5xFAD compared to WT. It is interesting to note that 5xFAD soluble APP do not differ from WT until 4 months of age, whereas a dramatic increase of membrane full length APP occurs as early as 2 months. This may indicate that the release and/or turnover of soluble forms of APP are tightly regulated in 5xFAD hippocampi. In contrast, a strong increase of CTFs at 2 months is in pace with the accumulation of their full-length precursor at the membrane. This finding may be particularly relevant, since the β-secretase derived fragment C99 has been described as an early neurotoxic metabolite of APP that accumulates much earlier than Aβ species (Lauritzen et al., 2012). In keeping with this idea, the increase of C99 levels at 2 months in our 5xFAD mice is in clear contrast with the finding that Aβ trimers could not be detected before 4 months of age. Thus, our data further support that early accumulation of C99 may trigger detrimental effects preceding those potentially caused by neurotoxic Aβ assemblies (i.e., trimers) at later time points.

### MT1-MMP displays amyloidogenic features

The *in vivo* data argue in favor of a functional link between the upregulation of MT1-MMP levels and amyloidosis. In support of this hypothesis the overexpression of MT1-MMP stimulated both C99 and Aβ production by HEKswe cells. Previous work using wild type HEK cells demonstrated that overexpression of both MT1-MMP and APP results in the generation of several soluble APP fragments with undetermined functional consequences (Ahmad et al., 2006). In line with our data, one possible consequence of APP cleavage by MT1-MMP would be to stimulate the generation of C99 and then Aβ production by γ-secretase. However, none of the soluble APP fragments identified in the Ahmad et al. (2006) study resulted from unambiguous β-secretase cleavage. It is thus unlikely that C99 results from at direct β-site cleavage of APP by MT1-MMP, and rather points out to a modulatory effect of this MMP on the processing of APP by β-secretase. Our data provide evidence for a new role of MT1-MMP as a pro-amyloidogenic enzyme. However, it has been shown that MMP overexpression in COS cells, as well as soluble MT1-MMP are both able to degrade exogenous synthetic Aβ (Liao and Van Nostrand, 2010). Altogether, these findings suggest the possibility of a dual role for MT1-MMP that might depend on the location and the way APP and Aβ are processed. The MT1-MMP-mediated increase of C99 in HEKswe cells supports an intracellular action of MT1-MMP, possibly stimulating amyloidogenesis *via* β-secretase activity and/or modifying APP trafficking that needs to be further evaluated. This could reflect the situation in 5xFAD neuronal cells where MT1-MMP and APP levels increase with age, which does not preclude a putative intrinsic capacity of MT1-MMP to degrade exogenous Aβ. The latter would be more physiologically compatible with situations where MT1-MMP would be produced by surrounding glial cells that could degrade Aβ released by neurons.

In full agreement with the literature on Aβ proteolysis (see De Strooper, 2010; Rivera et al., 2010 for review), overexpressing MMP-2 in our cell system nearly abolished Aβ production. This occurred without affecting C99 levels, in clear contrast with the effects of MT1-MMP. Both MMPs have been described to interact with TIMP-2 in a ternary complex where MT1-MMP activates MMP-2 (Strongin et al., 1995). In the present case, MMP-2 and MT1-MMP seem to work independently, at least as far as the generation of C99 and Aβ is concerned.

Our data provide evidence that the age of 4 months signals a time for overt accumulation of Aβ trimers and C99. This sudden activation of APP metabolism occurs at a prodromal-like phase of the pathology, where the first cognitive deficits are observed in 5xFAD males tested in a hippocampal-dependent olfactory task (Girard et al., 2014), and it is maintained over the symptomatic phase (6 months). Early accumulation (2–4 months) of Aβ 3-42 pyroglutamate, a highly toxic species of Aβ, and axonal swelling have also been reported in the hippocampus of 5xFAD mice (Jawhar et al., 2012; Moon et al., 2012). Together, these data highlight the interest of studying the physiopathology of the hippocampus in the 5xFAD mouse model, and provide further molecular support for the impairment of hippocampal synaptic transmission reported in 5xFAD mice (Kimura and Ohno, 2009; Crouzin et al., 2013). Our study provides the first evidence that different MMPs involved in APP/Aβ metabolism are differentially regulated in a spatio-temporal manner in the 5xFAD murine model of AD. Such upregulation is consistent with the putative role of MMP-2 and MMP-9 as Aβ-degrading enzymes, which could locally exert their actions once released by reactive astrocytes in the vicinity of plaques. Most interestingly, our data support the idea that MT1-MMP stimulates APP metabolism and amyloidogenesis. Although the *in vivo* data do not prove *per se* a functional link between the increase of active MT1-MMP and Aβ accumulation, these findings, together with our *in vitro* results, should set the basis to further validate a potential pro-amyloidogenic role of MT1-MMP and characterize the molecular mechanisms at stake.

## Authors and contributors

Nathalie A. Py contributed to most experiments, analyzed and formatted the data, and helped with manuscript writing. Amandine E. Bonnet contributed to *in vivo* and *in vitro* experiments and critically revised the manuscript. Anne Bernard and Eliane Charrat helped with the *in vivo* experiments. Yannick Marchalant helped with *in vivo* experiments and critically revised the manuscript. Frédéric Checler provided the HEKswe cells and critically revised the manuscript. Michel Khrestchatisky helped in the conception and design of the work and critically revised the manuscript. Kévin Baranger conceived and supervised the work, analyzed the biochemical data and co-wrote the manuscript. Santiago Rivera conceived and supervised the work, analyzed the *in vivo* data and co-wrote the manuscript.

## Conflict of interest statement

The authors declare that the research was conducted in the absence of any commercial or financial relationships that could be construed as a potential conflict of interest.

## References

[B1] Ahmad M. Takino T. Miyamori H. Yoshizaki T. Furukawa M. Sato H. (2006). Cleavage of amyloid-beta precursor protein (APP) by membrane-type matrix metalloproteinases. J. Biochem. 139, 517–526 10.1093/jb/mvj054 16567416

[B2] Backstrom J. R. Lim G. P. Cullen M. J. Tökés Z. A. (1996). Matrix metalloproteinase-9 (MMP-9) is synthesized in neurons of the human hippocampus and is capable of degrading the amyloid-beta peptide (1–40). J. Neurosci. 16, 7910–7919 8987819 10.1523/JNEUROSCI.16-24-07910.1996 PMC6579235

[B3] Buggia-Prevot V. Sevalle J. Rossner S. Checler F. (2008). NFkappaB-dependent control of BACE1 promoter transactivation by Abeta42. J. Biol. Chem. 283, 10037–10047 10.1074/jbc.M706579200 18263584

[B4] Candelario-Jalil E. Yang Y. Rosenberg G. A. (2009). Diverse roles of matrix metalloproteinases and tissue inhibitors of metalloproteinases in neuroinflammation and cerebral ischemia. Neuroscience 158, 983–994 10.1016/j.neuroscience.2008.06.025 18621108 PMC3584171

[B5] Chaillan F. A. Rivera S. Marchetti E. Jourquin J. Werb Z. Soloway P. D. (2006). Involvement of tissue inhibition of metalloproteinases-1 in learning and memory in mice. Behav. Brain Res. 173, 191–198 10.1016/j.bbr.2006.06.020 16860884 PMC2659720

[B6] Chami L. Checler F. (2012). BACE1 is at the crossroad of a toxic vicious cycle involving cellular stress and β-amyloid production in Alzheimer’s disease. Mol. Neurodegener. 7:52 10.1186/1750-1326-7-52 23039869 PMC3507664

[B7] Chevallier N. Jiracek J. Vincent B. Baur C. P. Spillantini M. G. Goedert M. (1997). Examination of the role of endopeptidase 3.4.24.15 in A beta secretion by human transfected cells. Br. J. Pharmacol. 121, 556–562 10.1038/sj.bjp.0701151 9179400 PMC1564707

[B8] Cleary J. P. Walsh D. M. Hofmeister J. J. Shankar G. M. Kuskowski M. A. Selkoe D. J. (2005). Natural oligomers of the amyloid-beta protein specifically disrupt cognitive function. Nat. Neurosci. 8, 79–84 10.1038/nn1372 15608634

[B9] Crouzin N. Baranger K. Cavalier M. Marchalant Y. Cohen-Solal C. Roman F. S. (2013). Area-specific alterations of synaptic plasticity in the 5XFAD mouse model of Alzheimer’s disease: dissociation between somatosensory cortex and hippocampus. PLoS One 8:e74667 10.1371/journal.pone.0074667 24069328 PMC3775744

[B10] Dafnis I. Tzinia A. K. Tsilibary E. C. Zannis V. I. Chroni A. (2012). An apolipoprotein E4 fragment affects matrix metalloproteinase 9, tissue inhibitor of metalloproteinase 1 and cytokine levels in brain cell lines. Neuroscience 210, 21–32 10.1016/j.neuroscience.2012.03.013 22445724 PMC3358542

[B11] Deb S. Wenjun Zhang J. Gottschall P. E. (2003). Beta-amyloid induces the production of active, matrix-degrading proteases in cultured rat astrocytes. Brain Res. 970, 205–213 10.1016/s0006-8993(03)02344-8 12706262

[B12] De Strooper B. (2010). Proteases and proteolysis in Alzheimer disease: a multifactorial view on the disease process. Physiol. Rev. 90, 465–494 10.1152/physrev.00023.2009 20393191

[B13] Fanjul-Fernández M. Folgueras A. R. Cabrera S. López-Otín C. (2010). Matrix metalloproteinases: evolution, gene regulation and functional analysis in mouse models. Biochim. Biophys. Acta 1803, 3–19 10.1016/j.bbamcr.2009.07.004 19631700

[B14] Giannoni P. Gaven F. de Bundel D. Baranger K. Marchetti-Gauthier E. Roman F. S. (2013). Early administration of RS 67333, a specific 5-HT4 receptor agonist, prevents amyloidogenesis and behavioral deficits in the 5XFAD mouse model of Alzheimer’s disease. Front. Aging Neurosci. 5:96 10.3389/fnagi.2013.00096 24399967 PMC3871961

[B15] Girard S. D. Baranger K. Gauthier C. Jacquet M. Bernard A. Escoffier G. (2013). Evidence for early cognitive impairment related to frontal cortex in the 5XFAD mouse model of Alzheimer’s disease. J. Alzheimers Dis. 33, 781–796 10.3233/JAD-2012-120982 23042214

[B16] Girard S. D. Jacquet M. Baranger K. Migliorati M. Escoffier G. Bernard A. (2014). Onset of hippocampus-dependent memory impairments in 5XFAD transgenic mouse model of Alzheimer’s disease. Hippocampus 24, 762–772 10.1002/hipo.22267 24596271

[B17] Gonthier B. Nasarre C. Roth L. Perraut M. Thomasset N. Roussel G. (2007). Functional interaction between matrix metalloproteinase-3 and semaphorin-3C during cortical axonal growth and guidance. Cereb. Cortex 17, 1712–1721 10.1093/cercor/bhl082 17021275

[B18] Hardy J. A. Higgins G. A. (1992). Alzheimer’s disease: the amyloid cascade hypothesis. Science 256, 184–185 10.1126/science.1566067 1566067

[B19] Higashi S. Miyazaki K. (2003). Novel processing of beta-amyloid precursor protein catalyzed by membrane type 1 matrix metalloproteinase releases a fragment lacking the inhibitor domain against gelatinase A. Biochemistry 42, 6514–6526 10.1021/bi020643m 12767235

[B20] Hongpaisan J. Sun M. K. Alkon D. L. (2011). PKC ε activation prevents synaptic loss, Aβ elevation and cognitive deficits in Alzheimer’s disease transgenic mice. J. Neurosci. 31, 630–643 10.1523/JNEUROSCI.5209-10.2011 21228172 PMC6623433

[B21] Hu J. Van den Steen P. E. Sang Q. X. Opdenakker G. (2007). Matrix metalloproteinase inhibitors as therapy for inflammatory and vascular diseases. Nat. Rev. Drug Discov. 6, 480–498 10.1038/nrd2308 17541420

[B22] Imai K. Ohuchi E. Aoki T. Nomura H. Fujii Y. Sato H. (1996). Membrane-type matrix metalloproteinase 1 is a gelatinolytic enzyme and is secreted in a complex with tissue inhibitor of metalloproteinases 2. Cancer Res. 56, 2707–2710 8665498

[B23] Jawhar S. Trawicka A. Jenneckens C. Bayer T. A. Wirths O. (2012). Motor deficits, neuron loss and reduced anxiety coinciding with axonal degeneration and intraneuronal Aβ aggregation in the 5XFAD mouse model of Alzheimer’s disease. Neurobiol. Aging 33, 196.e29–196.e40 10.1016/j.neurobiolaging.2010.05.027 20619937

[B24] Jourquin J. Tremblay E. Bernard A. Charton G. Chaillan F. A. Marchetti E. (2005). Tissue inhibitor of metalloproteinases-1 (TIMP-1) modulates neuronal death, axonal plasticity and learning and memory. Eur. J. Neurosci. 22, 2569–2578 10.1111/j.1460-9568.2005.04426.x 16307599

[B25] Jourquin J. Tremblay E. Décanis N. Charton G. Hanessian S. Chollet A. M. (2003). Neuronal activity-dependent increase of net matrix metalloproteinase activity is associated with MMP-9 neurotoxicity after kainate. Eur. J. Neurosci. 18, 1507–1517 10.1046/j.1460-9568.2003.02876.x 14511330

[B26] Jung S. S. Zhang W. Van Nostrand W. E. (2003). Pathogenic A beta induces the expression and activation of matrix metalloproteinase-2 in human cerebrovascular smooth muscle cells. J. Neurochem. 85, 1208–1215 10.1046/j.1471-4159.2003.01745.x 12753080

[B27] Kaliszewska A. Bijata M. Kaczmarek L. Kossut M. (2012). Experience-dependent plasticity of the barrel cortex in mice observed with 2-DG brain mapping and c-Fos: effects of MMP-9 KO. Cereb. Cortex 22, 2160–2170 10.1093/cercor/bhr303 22021911

[B28] Karran E. Mercken M. De Strooper B. (2011). The amyloid cascade hypothesis for Alzheimer’s disease: an appraisal for the development of therapeutics. Nat. Rev. Drug Discov. 10, 698–712 10.1038/nrd3505 21852788

[B29] Kimura R. Ohno M. (2009). Impairments in remote memory stabilization precede hippocampal synaptic and cognitive failures in 5XFAD Alzheimer mouse model. Neurobiol. Dis. 33, 229–235 10.1016/j.nbd.2008.10.006 19026746 PMC2741400

[B30] Langenfurth A. Rinnenthal J. L. Vinnakota K. Prinz V. Carlo A. S. Stadelmann C. (2013). Membrane-type 1 metalloproteinase is upregulated in microglia/brain macrophages in neurodegenerative and neuroinflammatory diseases. J. Neurosci. Res. 92, 275–286 10.1002/jnr.23288 24323769

[B31] Lauritzen I. Pardossi-Piquard R. Bauer C. Brigham E. Abraham J. D. Ranaldi S. (2012). The β-secretase-derived C-terminal fragment of βAPP, C99, but not Aβ, is a key contributor to early intraneuronal lesions in triple-transgenic mouse hippocampus. J. Neurosci. 32, 16243–16255 10.1523/JNEUROSCI.2775-12.2012 23152608 PMC5019353

[B32] LePage R. N. Fosang A. J. Fuller S. J. Murphy G. Evin G. Beyreuther K. (1995). Gelatinase a possesses a beta-secretase-like activity in cleaving the amyloid protein precursor of Alzheimer’s disease. FEBS Lett. 377, 267–270 10.1016/0014-5793(95)01358-x 8543065

[B33] Lesné S. Koh M. T. Kotilinek L. Kayed R. Glabe C. G. Yang A. (2006). A specific amyloid-beta protein assembly in the brain impairs memory. Nature 440, 352–357 10.1038/nature04533 16541076

[B34] Liao M. C. Van Nostrand W. E. (2010). Degradation of soluble and fibrillar amyloid beta-protein by matrix metalloproteinase (MT1-MMP) in vitro. Biochemistry 49, 1127–1136 10.1021/bi901994d 20050683 PMC2819544

[B35] Mazzone M. Baldassarre M. Beznoussenko G. Giacchetti G. Cao J. Zucker S. (2004). Intracellular processing and activation of membrane type 1 matrix metalloprotease depends on its partitioning into lipid domains. J. Cell Sci. 117, 6275–6287 10.1242/jcs.01563 15561768

[B36] Meighan S. E. Meighan P. C. Choudhury P. Davis C. J. Olson M. L. Zornes P. A. (2006). Effects of extracellular matrix-degrading proteases matrix metalloproteinases 3 and 9 on spatial learning and synaptic plasticity. J. Neurochem. 96, 1227–1241 10.1111/j.1471-4159.2005.03565.x 16464240

[B37] Mizoguchi H. Takuma K. Fukuzaki E. Ibi D. Someya E. Akazawa K. H. (2009). Matrix metalloprotease-9 inhibition improves amyloid beta-mediated cognitive impairment and neurotoxicity in mice. J. Pharmacol. Exp. Ther. 331, 14–22 10.1124/jpet.109.154724 19587312

[B38] Moon M. Hong H. S. Nam D. W. Baik S. H. Song H. Kook S. Y. (2012). Intracellular amyloid-beta accumulation in calcium-binding protein-deficient neurons leads to amyloid-beta plaque formation in animal model of Alzheimer’s disease. J. Alzheimers Dis. 29, 615–628 10.3233/JAD-2011-111778 22269161

[B39] Nagy V. Bozdagi O. Matynia A. Balcerzyk M. Okulski P. Dzwonek J. (2006). Matrix metalloproteinase-9 is required for hippocampal late-phase long-term potentiation and memory. J. Neurosci. 26, 1923–1934 10.1523/jneurosci.4359-05.2006 16481424 PMC4428329

[B40] Oakley H. Cole S. L. Logan S. Maus E. Shao P. Craft J. (2006). Intraneuronal beta-amyloid aggregates, neurodegeneration and neuron loss in transgenic mice with five familial Alzheimer’s disease mutations: potential factors in amyloid plaque formation. J. Neurosci. 26, 10129–10140 10.1523/jneurosci.1202-06.2006 17021169 PMC6674618

[B41] Ogier C. Bernard A. Chollet A. M. L. E. Diguardher T. Hanessian S. Charton G. (2006). Matrix metalloproteinase-2 (MMP-2) regulates astrocyte motility in connection with the actin cytoskeleton and integrins. Glia 54, 272–284 10.1002/glia.20349 16845676

[B42] Ogier C. Creidy R. Boucraut J. Soloway P. D. Khrestchatisky M. Rivera S. (2005). Astrocyte reactivity to Fas activation is attenuated in TIMP-1 deficient mice, an in vitro study. BMC Neurosci. 6:68 10.1186/1471-2202-6-68 16316466 PMC1325973

[B43] Ohno M. Cole S. L. Yasvoina M. Zhao J. Citron M. Berry R. (2007). BACE1 gene deletion prevents neuron loss and memory deficits in 5XFAD APP/PS1 transgenic mice. Neurobiol. Dis. 26, 134–145 10.1016/j.nbd.2006.12.008 17258906 PMC1876698

[B44] Ould-yahoui A. Tremblay E. Sbai O. Ferhat L. Bernard A. Charrat E. (2009). A new role for TIMP-1 in modulating neurite outgrowth and morphology of cortical neurons. PLoS One 4:e8289 10.1371/journal.pone.0008289 20011518 PMC2788270

[B45] Rivera S. Khrestchatisky M. Kaczmarek L. Rosenberg G. A. Jaworski D. M. (2010). Metzincin proteases and their inhibitors, foes or friends in nervous system physiology? J. Neurosci. 30, 15337–15357 10.1523/JNEUROSCI.3467-10.2010 21084591 PMC3072038

[B46] Rivera S. Ogier C. Jourquin J. Timsit S. Szklarczyk A. Miller K. (2002). Gelatinase B and TIMP-1 are regulated in a cell- and time dependent manner in association with neuronal death and glial reactivity after global forebrain ischemia. Eur. J. Neurosci. 15, 19–32 10.1046/j.0953-816x.2001.01838.x 11860503

[B47] Rivera S. Tremblay E. Timsit S. Canals O. Ben-Ari Y. Khrestchatisky M. (1997). Tissue inhibitor of metalloproteinases-1 (TIMP-1) is differentially induced in neurons and astrocytes after seizures: evidence for developmental, immediate early gene and lesion response. J. Neurosci. 17, 4223–4235 9151739 10.1523/JNEUROSCI.17-11-04223.1997 PMC6573546

[B48] Roher A. E. Kasunic T. C. Woods A. S. Cotter R. J. Ball M. J. Fridman R. (1994). Proteolysis of A beta peptide from Alzheimer disease brain by gelatinase A. Biochem. Biophys. Res. Commun. 205, 1755–1761 10.1006/bbrc.1994.2872 7811262

[B49] Sbai O. Ferhat L. Bernard A. Gueye Y. Ould-Yahoui A. Thiolloy S. (2008). Vesicular trafficking and secretion of matrix metalloproteinases-2, -9 and tissue inhibitor of metalloproteinases-1 in neuronal cells. Mol. Cell. Neurosci. 39, 549–568 10.1016/j.mcn.2008.08.004 18817873

[B50] Sbai O. Ould-Yahoui A. Ferhat L. Gueye Y. Bernard A. Charrat E. (2010). Differential vesicular distribution and trafficking of MMP-2, MMP-9 and their inhibitors in astrocytes. Glia 58, 344–366 10.1002/glia.20927 19780201

[B51] Shankar G. M. Li S. Mehta T. H. Garcia-Munoz A. Shepardson N. E. Smith I. (2008). Amyloid-beta protein dimers isolated directly from Alzheimer’s brains impair synaptic plasticity and memory. Nat. Med. 14, 837–842 10.1038/nm1782 18568035 PMC2772133

[B52] Soreghan B. Kosmoski J. Glabe C. (1994). Surfactant properties of Alzheimer’s A beta peptides and the mechanism of amyloid aggregation. J. Biol. Chem. 269, 28551–28554 7961799

[B53] Strongin A. Y. Collier I. Bannikov G. Marmer B. L. Grant G. A. Goldberg G. I. (1995). Mechanism of cell surface activation of 72-kDa type IV collagenase. Isolation of the activated form of the membrane metalloprotease. J. Biol. Chem. 270, 5331–5338 10.1074/jbc.270.10.5331 7890645

[B54] Talamagas A. A. Efthimiopoulos S. Tsilibary E. C. Figueiredo-Pereira M. E. Tzinia A. K. (2007). Abeta(1–40)-induced secretion of matrix metalloproteinase-9 results in sAPPalpha release by association with cell surface APP. Neurobiol. Dis. 28, 304–315 10.1016/j.nbd.2007.07.016 17761425

[B55] Townsend M. Shankar G. M. Mehta T. Walsh D. M. Selkoe D. J. (2006). Effects of secreted oligomers of amyloid beta-protein on hippocampal synaptic plasticity: a potent role for trimers. J. Physiol. 572, 477–492 10.1113/jphysiol.2005.103754 16469784 PMC1779683

[B56] Walsh D. M. Klyubin I. Fadeeva J. V. Cullen W. K. Anwyl R. Wolfe M. S. (2002). Naturally secreted oligomers of amyloid beta protein potently inhibit hippocampal long-term potentiation in vivo. Nature 416, 535–539 10.1038/416535a 11932745

[B57] Yan P. Hu X. Song H. Yin K. Bateman R. J. Cirrito J. R. (2006). Matrix metalloproteinase-9 degrades amyloid-beta fibrils in vitro and compact plaques in situ. J. Biol. Chem. 281, 24566–24574 10.1074/jbc.m602440200 16787929

[B58] Yang Y. Estrada E. Y. Thompson J. F. Liu W. Rosenberg G. A. (2007). Matrix metalloproteinase-mediated disruption of tight junction proteins in cerebral vessels is reversed by synthetic matrix metalloproteinase inhibitor in focal ischemia in rat. J. Cereb. Blood Flow Metab. 27, 697–709 10.1038/sj.jcbfm.9600375 16850029

[B59] Yin K.-J. Cirrito J. R. Yan P. Hu X. Xiao Q. Pan X. (2006). Matrix metalloproteinases expressed by astrocytes mediate extracellular amyloid-beta peptide catabolism. J. Neurosci. 26, 10939–10948 10.1523/jneurosci.2085-06.2006 17065436 PMC6674654

